# Analysis of Structural Health Monitoring Data with Correlated Measurement Error by Bayesian System Identification: Theory and Application

**DOI:** 10.3390/s22207981

**Published:** 2022-10-19

**Authors:** He-Qing Mu, Xin-Xiong Liang, Ji-Hui Shen, Feng-Liang Zhang

**Affiliations:** 1 Key Laboratory of Earthquake Engineering and Engineering Vibration, Institute of Engineering Mechanics, China Earthquake Administration, Harbin 150080, China; 2 Key Laboratory of Earthquake Disaster Mitigation, Ministry of Emergency Management, Harbin 150080, China; 3 School of Civil Engineering and Transportation, State Key Laboratory of Subtropical Building Science, Guangzhou 510640, China; 4 School of Civil and Environmental Engineering, Harbin Institute of Technology, Shenzhen 518055, China

**Keywords:** Bayesian inference, correlation, measurement error, model class selection, structural health monitoring

## Abstract

Measurement error is non-negligible and crucial in SHM data analysis. In many applications of SHM, measurement errors are statistically correlated in space and/or in time for data from sensor networks. Existing works solely consider spatial correlation for measurement error. When both spatial and temporal correlation are considered simultaneously, the existing works collapse, as they do not possess a suitable form describing spatially and temporally correlated measurement error. In order to tackle this burden, this paper generalizes the form of correlated measurement error from spatial correlation only or temporal correlation only to spatial-temporal correlation. A new form of spatial-temporal correlation and the corresponding likelihood function are proposed, and multiple candidate model classes for the measurement error are constructed, including no correlation, spatial correlation, temporal correlation, and the proposed spatial-temporal correlation. Bayesian system identification is conducted to achieve not only the posterior probability density function (PDF) for the model parameters, but also the posterior probability of each candidate model class for selecting the most suitable/plausible model class for the measurement error. Examples are presented with applications to model updating and modal frequency prediction under varying environmental conditions, ensuring the necessity of considering correlated measurement error and the capability of the proposed Bayesian system identification in the uncertainty quantification at the parameter and model levels.

## 1. Introduction

Structural health monitoring (SHM), which is to use measured data to infer the health status of the monitored structure, has received tremendous attention over the last decades [1,2,3,4,5,6,7,8,9,10]. For a particular SHM problem, the model output reflects the corresponding parameterization, and measurement error is the discrepancy between the measured noisy output and model output. In SHM data analysis, as measurement error is non-negligible, it is crucial to select a suitable form and conduct system identification for the measurement error.

Deterministic inference methods typically construct the objective function as the generalized least squares under the assumption of the covariance matrix of measurement errors. Although the assumption of uncorrelated measurement error is widely adopted [11], it is found that inference can be improved by relaxing the uncorrelation assumption in some circumstances [12,13]. Probabilistic inference methods construct the objective function as the likelihood function in the frequentist approach or a posterior Probability Density Function (PDF) in the Bayesian approach [14,15,16,17]. Using Bayes’ Theorem, the posterior PDF, which is proportional to the prior PDF and the likelihood function, accounts for the uncertainty both in the prior knowledge as well as in the measurements. The construction of the likelihood requires selecting a probability model for measurement error. The joint probability distribution of the measurement error vector is related to the marginal probability distribution and the correlation function. The marginal probability distribution prescribes that each measurement error follows a univariate Gaussian distribution, according to the Principle of Maximum Entropy [18,19]. The correlation function characterizes the statistical correlation between any two distinct measurement errors.

In many applications of SHM, measurement errors are statistically correlated in space (especially for dense sensor grids), time (especially for high sampling frequencies), or both in space and time, for data from sensor networks [20]. For the purpose of considering the statistical correlation of measurement errors, different correlation models have been considered. For instance, McFarland and Mahadevan [12] chose temporally exponential correlation for model calibration of thermal problems. Cheung et al. [21] considered spatially exponential correlation for turbulence modeling. Papadimitriou and Lombaert [22] investigated the effect of spatially exponential correlation for optimal sensor placement. Simoen et al. [23] introduced different functions for spatial correlation, e.g., an exponential correlation function, a spherical correlation function, and an exponentially damped cosine correlation function. Mu and Yuen [24] considered identical correlation in sparse Bayesian learning for risk pattern recognition.

Based on a prescribed set of candidate model classes for the correlated measurement error, Bayesian model class selection can be performed. The most suitable/plausible model class of measurement error is the one possessing maximum posterior probability. Recently, Bayesian model class selection has been studied and developed for structural health monitoring [25,26,27,28,29], structural damage detection [30,31,32,33], and risk assessment [24,34,35]. In particular, Simoen et al. [23] solely considered spatial correlation for measurement error and performed Bayesian model class selection to select the most suitable/plausible model class of spatially correlated measurement error in model updating. When temporal correlation alone is considered, an extension of the existing works [12,21,22,23] should be made, not only introducing a suitable form describing temporally correlated measurement error, but also deriving the corresponding inferences. Furthermore, when both spatial and temporal correlation are considered simultaneously, i.e., spatial-temporal correlation, the existing works [12,21,22,23] collapse, as they do not possess a suitable form describing spatially and temporally correlated measurement error. In order to tackle this burden, this paper generalizes the form of correlated measurement error from spatial correlation only or temporal correlation only to spatial-temporal correlation. A new form of spatial-temporal correlation and the corresponding likelihood function are proposed, and multiple candidate model classes for the measurement error are constructed, including no correlation, spatial correlation, temporal correlation, and the proposed spatial-temporal correlation. Bayesian system identification is conducted to achieve not only the posterior probability density function (PDF) for the model parameters, but also the posterior probability of each candidate model class for selecting the most suitable/plausible model class for the measurement error.

In Section 2, the theory is presented, including candidate model classes of data with correlated measurement error, posterior probability density function for uncertain model parameters, and posterior probability for model class selection. In Section 3, examples are presented with applications for model updating and modal frequency prediction under varying environmental conditions.

## 2. Theory

### 2.1. Candidate Model Classes of Data with Correlated Measurement Error

Consider a structure monitored by a sensor network with $N_{o}$ observations at each time step. Let $Q_{n}(b|\bar{S})\in {\mathbb{R}}^{N_{o}}$ denote the model output at the n-th time step, which is parameterized by a structural model $\bar{S}$ with the unknown structural parameter vector b. For a SHM problem, the model output reflects the corresponding parameterization. For example, in structural model updating, $\bar{S}$ can represent a particular structural model with unknown stiffness and damping parameters b. As the measured data are always subject to measurement error, the measured noisy output at the n-th time step $Y_{n}\in {\mathbb{R}}^{N_{o}}$:

(1) $$Y_{n}=Q_{n}(b|\bar{S})+\varepsilon_{n}(\tau |\bar{E}),n=1,\ldots ,N_{T}$$

where $\varepsilon_{n}\left(\tau |\bar{E}\right)\in {\mathbb{R}}^{N_{o}}$ is the uncertain measurement error at the n-th time step, parameterized by a measurement error model $\bar{E}$ with the unknown measurement error parameter vector τ. By collecting data up to $N_{T}$ sampling time steps, the measured noisy output matrix is $Y\in {\mathbb{R}}^{N_{o}\times N_{T}}=\left[Y_{1},\ldots ,Y_{N_{T}}\right]$, the model output matrix is $Q\in {\mathbb{R}}^{N_{o}\times N_{T}}=\left[Q_{1},\ldots ,Q_{N_{T}}\right]$, and the uncertain measurement error matrix is $\varepsilon \in {\mathbb{R}}^{N_{o}\times N_{T}}=\left[\varepsilon_{1},\ldots ,\varepsilon_{N_{T}}\right]$.

In SHM, the selection of a mathematical model of correlated measurement error $\bar{E}$ directly affects the inference for the structural model $\bar{S}$ and unknown structural parameters b. The joint probability distribution of ε is related to two assumptions: the marginal probability distribution of $\varepsilon_{ij}$ and the correlation function. The marginal probability distribution of $\varepsilon_{ij}$, according to the Principle of Maximum Entropy [18,19], follows univariate Gaussian distribution with zero mean and unknown variance $\tau_{0}$. That is, $\varepsilon_{ij}\sim N\left[\varepsilon_{ij}|0,\tau_{0}\right]$. For one thing, the zero mean assumption is valid, as uncertain bias can be added to model output as another uncertain parameter. For another thing, the variance of measurement error is an uncertain parameter to be identified. The homogeneity variance model assumes the variances of the distinct measurement errors are identical, while the heterogeneous variance model assumes they are different. Further discussion on homogeneity and heterogeneous variance models can be found in [35,36]. The correlation function characterizes the statistical correlation between any two distinct measurement errors ($\varepsilon_{ij}$ and $\varepsilon_{i\prime j\prime }$). The following correlation models are introduced: (1) uncorrelated model (denoted as $E_{1}$); (2) identical correlation model [24] (denoted as $E_{2}$); (3) exponential correlation model [23] (denoted as $E_{3}$). Let $\rho_{ij}^{E_{k}}$ denote the correlation function of two variables ***i*** and ***j*** conditional on $E_{j}$, described as follows:


(2) $$\rho_{ij}^{E_{1}}=\delta_{ij}$$

(3) $$\rho_{ij}^{E_{2}}=\delta_{ij}+\left(1-\delta_{ij}\right)\tau_{\gamma }^{E_{2}}$$

(4) $$\rho_{ij}^{E_{3}}=\delta_{ij}+\left(1-\delta_{ij}\right)\exp\left(-\frac{\Delta_{ij}}{\tau_{\gamma }^{E_{3}}}\right)$$

where $\delta_{ij}$ is the Kronecker delta, and $\Delta_{ij}=\left|i-j\right|$ represents the distance between measurements *i* and *j*. Based on $\rho_{ij}^{E_{k}}$, the spatial and temporal correlations can be constructed. Let $E_{spat}=\{E_{spat,m}=E_{m}|m=1,\ldots \}$ and $E_{temp}=\{E_{temp,n}=E_{n}|n=1,\ldots \}$ denote the candidate model classes for the correlated measurement error of spatial correlation and temporal correlation, respectively. Accordingly, introduce spatial correlation matrix $L^{E_{spat,m}}\in {\mathbb{R}}^{N_{o}\times N_{o}}$ and temporal correlation matrix $L^{E_{temp,n}}\in {\mathbb{R}}^{N_{T}\times N_{T}}$ as follows:


(5) $$L_{ij}^{E_{spat,m}}=\rho_{ij}^{E_{m}},i,j=1,\ldots ,N_{o},m=1,2,3$$

(6) $$L_{ij}^{E_{temp,n}}=\rho_{ij}^{E_{n}},i,j=1,\ldots ,N_{T},n=1,2,3$$

It is worth noting that the existing works [12,21,22,23] are capable of considering either spatial correlation of Equation (5) only or temporal correlation of Equation (6) only, and they do not possess a suitable form describing spatially and temporally correlated measurement error. Here, a new form of spatial-temporal correlation is proposed as follows. Let $\rho_{\varepsilon_{ij},\varepsilon_{i\prime j\prime }}^{E_{spat,m}E_{temp,n}}$, $\rho_{ii\prime }^{E_{n}}$, and $\rho_{j\prime j\prime }^{E_{m}}$ denote the spatial-temporal correlation of $\varepsilon_{ij}$ and $\varepsilon_{i\prime j\prime }$ the temporal correlation between $\varepsilon_{ij}$ and $\varepsilon_{i\prime j}$, and the spatial correlation between $\varepsilon_{i\prime j}$ and $\varepsilon_{i\prime j\prime }$, respectively. Based on the property of correlation, the discrepancy between the spatial-temporal correlation and the product $\rho_{ii\prime }^{E_{n}}\rho_{j\prime j\prime }^{E_{m}}$ is bounded as follows: $-\sqrt{\left(1-{\left(\rho_{ii\prime }^{E_{n}}\right)}^{2})(1-{\left(\rho_{j\prime j\prime }^{E_{m}}\right)}^{2}\right)}\leq \rho_{\varepsilon_{ij},\varepsilon_{i\prime j\prime }}^{E_{spat,m}E_{temp,n}}-\rho_{ii\prime }^{E_{n}}\rho_{j\prime j\prime }^{E_{m}}\leq \sqrt{\left(1-{\left(\rho_{ii\prime }^{E_{n}}\right)}^{2})(1-{\left(\rho_{j\prime j\prime }^{E_{m}}\right)}^{2}\right)}$. When $\left|\rho_{ii\prime }^{E_{n}}\right|$ and $\left|\rho_{j\prime j\prime }^{E_{m}}\right|$ are closed to 1 (high correlation in time and space), the following approximation for the spatial-temporal correlation holds: $\rho_{\varepsilon_{ij},\varepsilon_{i\prime j\prime }}^{E_{spat,m}E_{temp,n}}\approx \rho_{ii\prime }^{E_{n}}\rho_{j\prime j\prime }^{E_{m}}$. Thus, the covariance matrix of the spatial-temporal correlated measurement error vector can be expressed as:

(7) $$\mathrm{cov}\left[\mathrm{vec}\left(\varepsilon \right),\mathrm{vec}\left(\varepsilon \right)\right]=\Sigma^{E_{spat,m}E_{temp,n}}=\tau_{0}L^{E_{temp,n}}\;⊗\;L^{E_{spat,m}}$$

where vec() is vectorization; ⊗ is the Kronecker product. When setting $L^{E_{temp,n}}$ or $L^{E_{spat,m}}$ to be an identity matrix, the proposed spatial-temporal correlation in Equation (7) degenerates to the spatial correlation only or temporal correlation only. That is, while existing works consider spatial correlation alone [12,21,22,23], this paper considers the special case of spatial-temporal correlation.

The universal set of candidate model classes for the correlated measurement error is $E=E_{spat}\times E_{temp}$, and the corresponding unknown parameter vectors of measurement error:


(8) $$\tau =\left\{\begin{array}{c} \left\{\tau_{0}\right\},\mathrm{if}E_{spat,m}=E_{spat,1},E_{temp,n}=E_{temp,1}\\ \left\{\tau_{0},\tau_{\gamma }^{E_{temp,n}}\right\},\mathrm{if}E_{spat,m}=E_{spat,1},E_{temp,n}\neq E_{temp,1}\\ \left\{\tau_{0},\tau_{\gamma }^{E_{spat,m}}\right\},\mathrm{if}E_{spat,m}\neq E_{spat,1},E_{temp,n}=E_{temp,1}\\ \left\{\tau_{0},\tau_{\gamma }^{E_{spat,m}},\tau_{\gamma }^{E_{temp,n}}\right\},\mathrm{if}E_{spat,m}\neq E_{spat,1},E_{temp,n}\neq E_{temp,1} \end{array}\right.$$

Finally, the universal set of candidate model classes for the system and correlated measurement error is $M=S\times E=\left\{M_{k},k=1,2,\ldots \right\}$. As the joint probability model of the measured noisy output matrix Y is an $N_{o}N_{T}$-dimensional normal distribution $N\left[\mathrm{vec}\left(Y\right)|\mathrm{vec}\left(Q\left(X,b|S_{i}\right)\right),\tau_{0}L^{E_{temp,n}}⊗L^{E_{spat,m}}\right]$, the proposed likelihood function of SHM dataset D with spatially and temporally correlated measurement error, which is conditional on $M_{k}$ with its associated parameter vector $\theta ={\left[b^{T},\tau^{T}\right]}^{T}$, can be expressed as:

(9) $$p\left(D|\theta ,M_{k}\right)={\left(2\pi \tau_{0}\right)}^{-\frac{N_{o}N_{T}}{2}}{\left|L^{E_{spat,m}}\right|}^{-\frac{1}{2}}{\left|L^{E_{temp,n}}\right|}^{-\frac{1}{2}}\exp\left[-\frac{1}{2\tau_{0}}\mathrm{tr}\left\{{\left(L^{E_{temp,n}}\right)}^{-1}{\left[Y-Q\left(X,b|S_{i}\right)\right]}^{T}{\left(L^{E_{spat,m}}\right)}^{-1}\left[Y-Q\left(X,b|S_{i}\right)\right]\right\}\right]$$

where tr is the trace of the matrix. When setting $L^{E_{temp,n}}$ or $L^{E_{spat,m}}$ to be an identity matrix, the proposed likelihood function for spatial-temporal correlation in Equation (9) degenerates to the traditional likelihood function for the spatial correlation only or temporal correlation only in the existing works [12,21,22,23]. Based on the proposed likelihood function, Bayesian system identification is rederived in the following parts.

### 2.2. Posterior Probability Density Function for Uncertain Model Parameters

According to Bayes’ theorem, the posterior PDF is [14]:

(10) $$p\left(\theta |D,M_{k}\right)\propto p\left(D|\theta ,M_{k}\right)p\left(\left.\theta \right|M_{k}\right)$$

where $p\left(\left.\theta \right|M_{k}\right)$ is the prior PDF, reflecting uncertainty introduced by modeling error at the parameter level based on the prior information. The form of the prior PDF can be determined using the Principle of Maximum Entropy [18] or the conjugate distribution. According to the Principle of Maximum Entropy, $N\left(\theta_{i}|\mu ,\nu^{2}\right)$ (normally distributed prior PDFs with mean μ and variance $\nu^{2}$) and $Gamma\left(\theta_{i}|\alpha ,\beta \right)$ (Gamma-distributed prior PDFs with the shape factor α and scale factor β) are appointed to $\theta_{i}\in \left(-\infty ,+\infty \right)$ and $\theta_{i}\in \left(0,+\infty \right)$, respectively. In the conjugate distribution, $\tau_{0}$ follows $Inv-Gamma\left(\tau_{0}|\alpha \prime ,\beta \prime \right)$ (Inverse Gamma-distributed prior PDFs with the shape factor α′ and scale factor β′). A rational way of determining the hyperparameters of the prior PDF can be achieved as follows. First, according to the previous information, prescribe the values of Maximum A Priori (*MAPr*) and coefficients of variation value (*COV*). Then, determine the hyperparameters based on the *MAPr* and *COV*. For $N\left(\theta_{i}|\mu ,\nu^{2}\right)$, μ=MAPr and ν=MAPr·COV; for $Gamma\left(\theta_{i}|\alpha ,\beta \right),\;\alpha >1$, $\alpha =1/{\left(COV\right)}^{2}$ and $\beta =MAPr/\left[1/{\left(COV\right)}^{2}-1\right]$; for $Inv-Gamma\left(\tau_{0}|\alpha \prime ,\beta \prime \right),\alpha \prime >2,\;\mathrm{solving}\;\mathrm{the}\;\mathrm{following}\;\mathrm{equations}:\;\alpha \prime ={\left(1/COV\right)}^{2}+2,\beta \prime =\left[1/{\left(COV\right)}^{2}+3\right]MAPr$.

The posterior PDF $p\left(\theta |D,M_{k}\right)$ is dominated by $p\left(D|\theta ,M_{k}\right)$, given that the amount of datasets is large and $p\left(\left.\theta \right|M_{k}\right)$ is relatively flat. Denote $L\left(\theta |D,M_{k}\right)=-\ln p\left(\theta |D,M_{k}\right)$. The Maximum A Posteriori (*MAP*) estimate is [16]:

(11) $$\hat{\theta }=\underset{\theta }{\arg\min}L\left(\theta |D,M_{k}\right)\approx \underset{\theta }{\arg\min}\left[-\ln p\left(D|\theta ,M_{k}\right)\right]$$

where

(12) $$\begin{array}{c} -\ln p\left(D|\theta ,M_{k}\right)=\frac{N_{o}N_{T}}{2}\ln\left(2\pi \tau_{0}\right)+\frac{1}{2}\ln\left(\left|L^{E_{spat,m}}\right|\right)+\frac{1}{2}\ln\left(\left|L^{E_{temp,n}}\right|\right)\\ \;+\frac{1}{2\tau_{0}}\mathrm{tr}\left\{{\left(L^{E_{temp,n}}\right)}^{-1}{\left[Y-Q\left(X,b|S_{i}\right)\right]}^{T}{\left(L^{E_{spat,m}}\right)}^{-1}\left[Y-Q\left(X,b|S_{i}\right)\right]\right\} \end{array}$$

The posterior covariance matrix of the parameters $\Sigma_{\theta }$ is related to the local curvature of $L\left(\theta |D,M_{k}\right)$, which can be described by the hessian Matrix $H\left(L\left(\theta |D,M_{k}\right)\right)$ as follows:

(13) $$H\left(L\left(\theta |D,M_{k}\right)\right)=\left[\begin{array}{ccc} \frac{\partial^{2}L}{\partial \theta_{1}^{2}} & \cdots & \frac{\partial^{2}L}{\partial \theta_{1}\partial \theta_{n}}\\ \vdots & \ddots & \vdots \\ \frac{\partial^{2}L}{\partial \theta_{1}\partial \theta_{n}} & \ldots & \frac{\partial^{2}L}{\partial \theta_{n}^{2}} \end{array}\right]$$

In the globally identifiable case [4], the posterior PDF $p\left(\theta |D,M_{k}\right)$ can be well approximated by the multivariate normal distribution $N\left[\theta |\hat{\theta },{\left[H\left(L\left(\hat{\theta }|D,M_{k}\right)\right)\right]}^{-1}\right]$.

### 2.3. Posterior Probability for Model Class Selection

The purpose of model class selection is to select the most suitable model class based on the universal set of candidate model classes for the system; correlated measurement error is $M=S\times E=\left\{M_{k},k=1,2,\ldots \right\}$. Note that the determination of the universal set of candidate model classes M reflects uncertainty introduced by modeling error at the model level based on the prior information.

According to Bayes’ theorem, the posterior probability of model class $M_{k}$ is [16,30]:

(14) $$P\left(M_{k}|D\right)=\frac{p\left(D|M_{k}\right)P\left(M_{k}\right)}{\sum_{k}p\left(D|M_{k}\right)P\left(M_{k}\right)}$$

where $P\left(M_{k}|D\right)$ is the posterior probability of $M_{k}$ (also called the plausibility of $M_{k}$); $p\left(D|M_{k}\right)$ is the evidence (also called the marginal likelihood); $P\left(M_{k}\right)$ is the prior probability of $M_{k}$. When $P\left(M_{k}\right)$ is selected to be the discrete uniform distribution, the most suitable/plausible model class is $\hat{M}$, possessing the largest evidence:

(15) $$\hat{M}=\underset{M_{k}}{\arg\;\max}P\left(M_{k}|D\right)=\underset{M_{k}}{\arg\;\max}p\left(D|M_{k}\right)$$

In the globally identifiable case, $p\left(D|M_{k}\right)$ can be well approximated as [30]:

(16) $$p\left(D|M_{k}\right)\approx p\left(D|\hat{\theta },M_{k}\right)O_{k}$$

where $p\left(D|\hat{\theta },M_{k}\right)$ is the likelihood function evaluated at $\hat{\theta }$; $O_{k}$ is the Ockham factor [15,16,30]:

(17) $$O_{k}=p\left(\left.\hat{\theta }\right|M_{k}\right){\left(2\pi \right)}^{\frac{N_{\theta }}{2}}{\left|H\left(L\left(\hat{\theta }|D,M_{k}\right)\right)\right|}^{-\frac{1}{2}}$$

It can be seen that the optimal model $\hat{M}$ should balance between the model fitting capability (quantified by $p(D|\hat{\theta },M_{k})$) and the model robustness (quantified by $O_{k}$). Recall two correlation functions $\rho_{ij}^{E_{1}}$ and $\rho_{ij}^{E_{2}}$ of Equations (2) and (3), $\rho_{ij}^{E_{1}}$ can be viewed as a simplified case of $\rho_{ij}^{E_{2}}$ given that $\tau_{\gamma }^{E_{2}}=0$. On the one hand, if the model fitting capability is adopted for model class selection, $\rho_{ij}^{E_{2}}$ is superior to $\rho_{ij}^{E_{1}}$. On the other hand, if the model robustness is adopted for model class selection, $\rho_{ij}^{E_{1}}$ is superior to $\rho_{ij}^{E_{2}}$. From a Bayesian point of view, the model fitting capability and the model robustness should be considered simultaneously for selecting the most suitable/plausible correlation models of measurement error.

## 3. Illustrative Example

Two examples are presented with applications for model updating and modal frequency prediction under varying environmental condition. In each example, the SHM data with correlated measurement errors are analyzed by the proposed Bayesian system identification. It is worth noting that the true correlation model and the associated parameters of measurement error, as well as the structural parameters, are unknown during the process of system identification.

### 3.1. Application to Model Updating

Model updating has received tremendous attention in many science and engineering fields. In SHM, Bayesian system identification has been studied and developed to conduct uncertainty quantification [37,38,39,40,41,42,43]. This example demonstrates the analysis of SHM data with correlated measurement errors in model updating. Consider a five-story shear building subjected to ground accelerations. From the first to the fifth floor, the nominal values of mass are 270 ton, 260 ton, 250 ton, 240 ton, and 220 ton, respectively; the nominal values of stiffness are 250 MN/m, 225 MN/m, 200 MN/m, 175 MN/m, and 150 MN/m, respectively. The Rayleigh damping model is adopted and the damping ratios of the first and third modes are 5%. The base excitation is El Centro earthquake. In the following section, two cases of structural health status are considered: (1) no damage case, the structure is undamaged, so all structural parameters are equal to their nominal values; (2) damaged case, stiffnesses of the 1st and 3th stories are both reduced by 20%, while that of other floors remains unchanged. Accelerations of 1st, 3th, and 5th floors are measured. The measurement error follows a zero-mean multivariate normal distribution with the covariance matrix as $\Sigma^{E_{spat,2}E_{temp,2}}$. That is, both spatial and temporal correlations follow the identical correlation model ($E_{2}$). The measurement noise level is taken to be 15% rms of the noise-free responses of the top floor. Note that the true correlation model and the associated parameters of measurement error, as well as the structural parameters, are unknown during the process of system identification.

The candidate model class of the structure is introduced. The unknown stiffness matrix K and damping matrix L are parametrized in the structural model class $S_{1}$:

(18) $$K\left(b^{K}|S_{1}\right)=\overset{N_{K}^{S_{1}}}{\underset{j=1}{\sum }}b_{j}^{K}{\tilde{K}}_{j}^{S_{i}};L\left(b^{L}|S_{1}\right)=\overset{N_{L}^{S_{1}}}{\underset{j=1}{\sum }}b_{j}^{L}{\tilde{L}}_{j}^{S_{i}}$$

where $b^{K}={\left[b_{1}^{K},\ldots ,b_{N_{K}^{S_{1}}}^{K}\right]}^{T}$ and $b^{L}={\left[b_{1}^{L},\ldots ,b_{N_{L}^{S_{1}}}^{L}\right]}^{T}$ are the uncertain stiffness parameter vector and uncertain damping parameter vector; ${\tilde{K}}_{j}^{S_{i}\;},\;j=1,\ldots ,N_{K}^{S_{i}},$ and ${\tilde{L}}_{j}^{S_{i}},\;j=1,\ldots ,N_{K}^{S_{i}},$ are the prescribed nominal stiffness and damping submatrices. The damage level of a substructure can be reflected through the reduction of the corresponding stiffness parameter. For example, 5% reduction of a stiffness parameter indicates 5% stiffness loss of the corresponding substructure. The candidate model classes for the measurement error are introduced as follows: (1) $E_{spat,1}E_{temp,1}$ ($E_{spat,1}=E_{1}$ and $E_{temp,1}=E_{1}$) does not consider any correlation spatially or temporally; (2) $E_{spat,2}E_{temp,2}$ ($E_{spat,2}=E_{2}$ and $E_{temp,2}=E_{2}$) considers that spatial and temporal correlations are both identical correlations; (3) $E_{spat,3}E_{temp,3}$ ($E_{spat,3}=E_{3}$ and $E_{temp,3}=E_{3}$) considers that spatial and temporal correlations are both exponential correlations ($E_{3}$). Finally, the universal set of all candidate model classes is $M=\{S_{1}E_{spat,1}E_{temp,1}$,$S_{1}E_{spat,2}E_{temp,2}$,$S_{1}E_{spat,3}E_{temp,3}\}$. It is worth noting that because both $S_{1}E_{spat,2}E_{temp,2}$ and $S_{1}E_{spat,3}E_{temp,3}$ correspond to the spatial-temporal correlation, the existing works [12,21,22,23] are incapable of handling these two candidate model classes. Table 1 shows the prior PDF of parameters.

**Figure 1:**
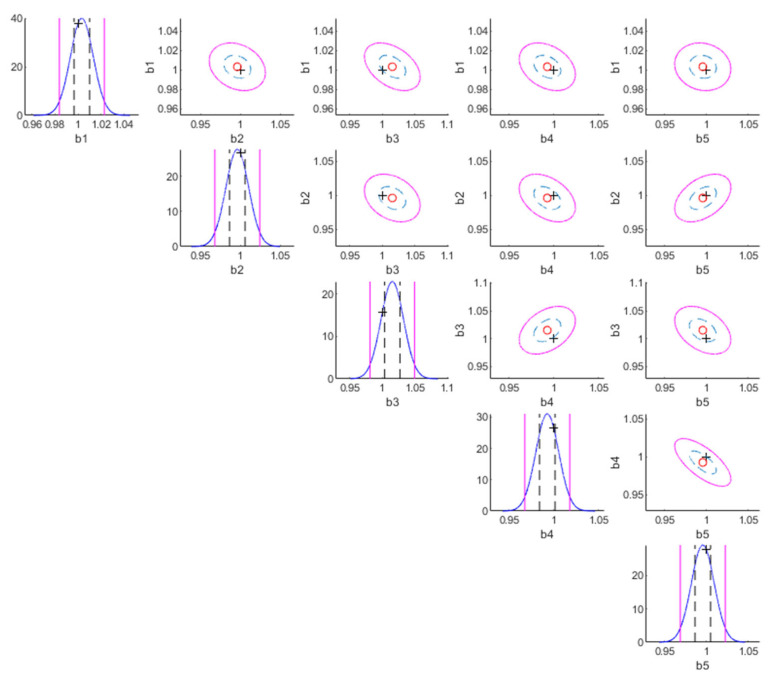
Contour plot of the posterior PDF of the substructure parameters of the most plausible model class (no damage case).

**Figure 2:**
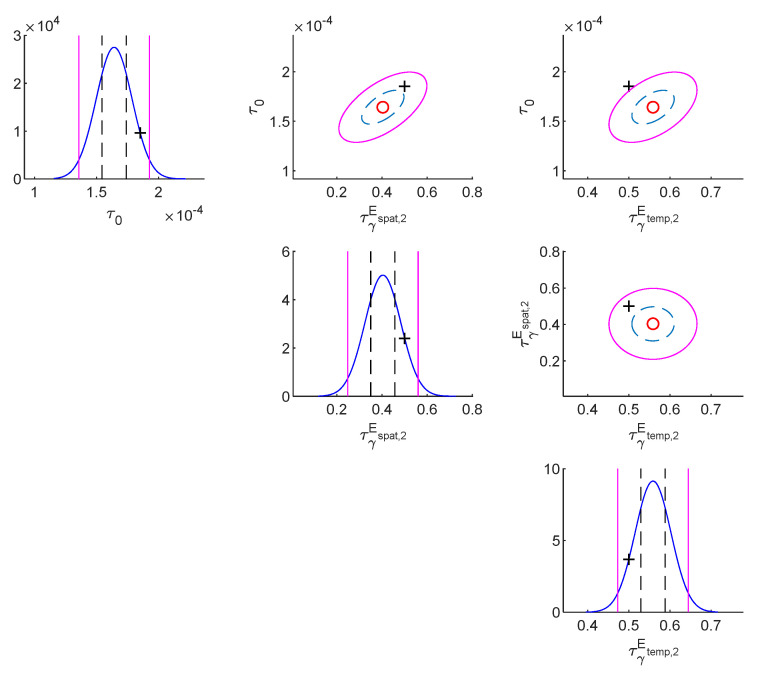
Contour plot of the posterior PDF of the measurement error parameters of the most plausible model class (no damage case).

The following are the results for the no damage case. Table 2 shows model class selection results (no damage case). The results of the most plausible class are in red color. For the log-likelihood, $S_{1}E_{spat,2}E_{temp,2}$ is superior. This is anticipated because the correlation pattern of $S_{1}E_{spat,2}E_{temp,2}$ is the same as the true correlation of measurement error. For the log-Ockham factor, $S_{1}E_{spat,1}E_{temp,1}$ is superior because the simplest parametrization possesses the highest robustness. Because the posterior model probability is the balance between the likelihood and Ockham factor, the optimal model class is $S_{1}E_{spat,2}E_{temp,2}$, which is identical to the true model class of measurement error. Table 3 shows parameter identification results (no damage case). Based on the capability of Bayesian inference for uncertainty quantification at the parameter level, both the *MAP* estimates and posterior standard deviations are shown. Compared to the true values of parameters, the *MAP* estimates of the most plausible class ($S_{1}E_{spat,2}E_{temp,2}$) are outperformed, and the corresponding posterior standard deviations of it are smaller than those of other model classes. Figure 1 shows the contour plot of the posterior PDF of the substructure parameters of the most plausible model class (no damage case). The true value, *MAP* estimate, 50% confidential interval, and 95% confidential interval are represented by “+”, “o”, dashed line, and solid line, respectively. The true values of the story stiffnesses are within 50% confidential intervals. Figure 2 shows the contour plots of the posterior PDF of the measurement error parameters of the most plausible model class (no damage case). The true values of the measurement error parameters are within the 95% confidential interval.

**Figure 3:**
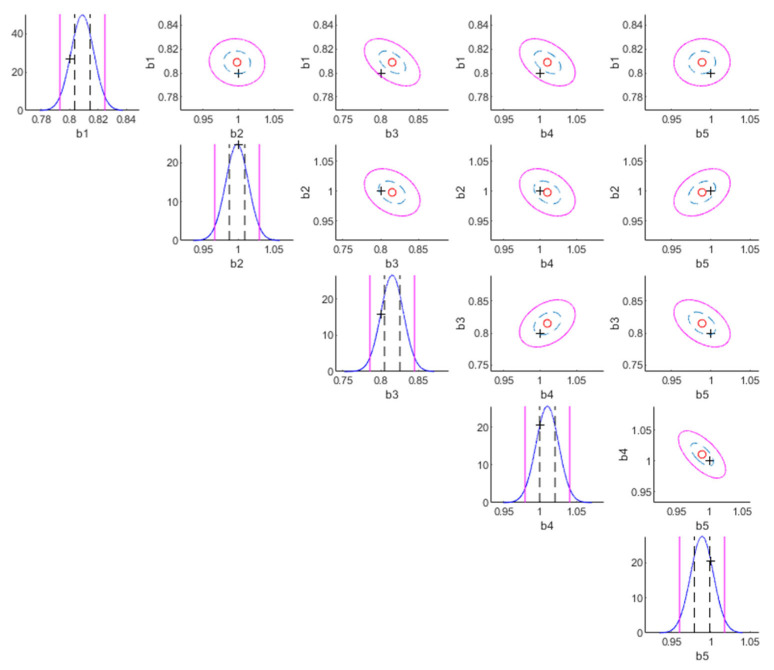
Contour plot of the posterior PDF of the substructure parameters of the most plausible model class (damaged case).

**Figure 4:**
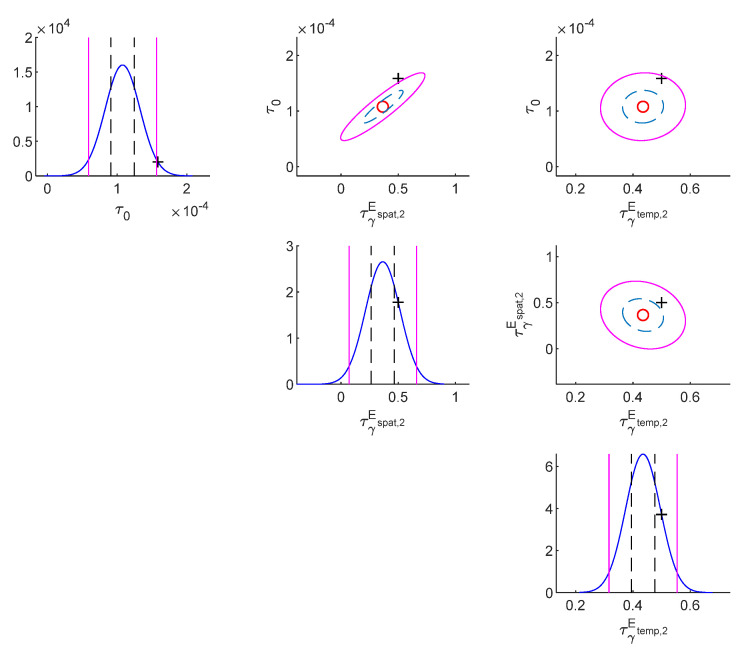
Contour plot of the posterior PDF of the measurement error parameters of the most plausible model (damaged case).

The following are the results for the damaged case. Table 4 shows model class selection results (damaged case). It can be anticipated that the true model class $S_{1}E_{spat,2}E_{temp,2}$ is selected because it is the best model balancing the likelihood and the Ockham factor. Table 5 shows parameter identification results (damaged case). The most plausible class ($S_{1}E_{spat,2}E_{temp,2}$) not only successfully detects stiffness reductions at the 1st and 3th floors, but also possesses the smallest posterior standard deviations. Figure 3 and Figure 4 show that both the true values of the story stiffnesses and measurement error parameters are within 50% confidential intervals and 95% confidential intervals, respectively.

The conclusions are as follows: (1) It is essential to select the most plausible model of measurement errors based on a prescribed set of candidate model classes characterizing spatially and temporally correlated measurement error; (2) over-simplified or over-complicated model class for the correlated measurement error in model updating degrades the identification result in terms of both the optimal value and uncertainty; (3) the proposed Bayesian system identification is capable of detecting structural damage with a satisfying precision level without knowing the true correlation model and the associated parameters.

### 3.2. Application to Modal Frequency Prediction under Varying Environmental Conditions

**Figure 5:**
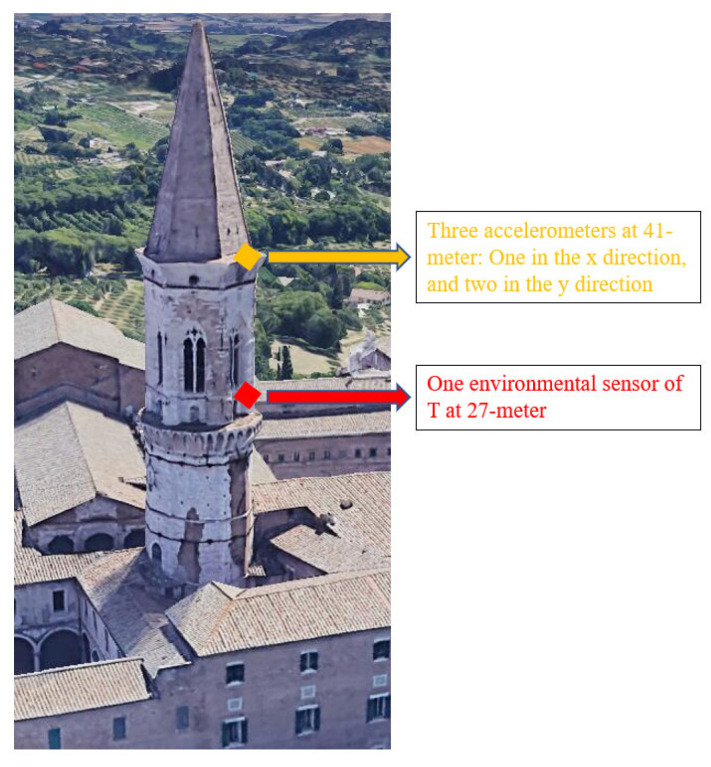
Side view (map data ©2022 Google) and sensor configuration.

This example demonstrates the analysis of SHM data with correlated measurement error in modal frequency prediction under varying environmental conditions. The monitored tower is located in Perugia, Italy. Figure 5 shows the side view and sensor configuration. One environmental sensor at 27 m and three accelerometers at 41 m were installed to obtain the temperature and modal parameters, respectively. Detailed information can be found in [44]. In the paper, a regression model is proposed to predict modal frequencies based on temperature:

(19) $$Q_{n}(b|\bar{S})=\left[\begin{array}{c} {\bar{\omega }}_{x1,n}\\ {\bar{\omega }}_{y1,n}\\ \begin{array}{c} {\bar{\omega }}_{y2,n}\\ {\bar{\omega }}_{y3,n} \end{array} \end{array}\right]=\left[\begin{array}{c} b_{x1,1}\\ b_{y1,1}\\ \begin{array}{c} b_{y2,1}\\ b_{y3,1} \end{array} \end{array}\right]+\left[\begin{array}{c} b_{x1,2}\\ b_{y1,2}\\ \begin{array}{c} b_{y2,2}\\ b_{y3,2} \end{array} \end{array}\right]\cdot 10^{-3}\cdot T_{n}$$

where $T_{n}$ is *n*-th input data point corresponding to the air temperature in the belfry outdoor of 27 m height and southern orientation; $\omega_{x1,n}$ are the *n*-th output data vector corresponding to the frequency of the 1st bending mode in the x direction; $\omega_{y1,n}$, $\omega_{y2,n}$, and $\omega_{y3,n}$ are the *n*-th output data vector corresponding to the frequencies of the 1st, 2nd, and 3rd bending modes in the y direction, respectively. The measured noisy output $Y_{n}$ is:

(20) $$Y_{n}=Q_{n}(b|\bar{S})+\varepsilon_{n}(\tau |\bar{E})$$

**Figure 6:**
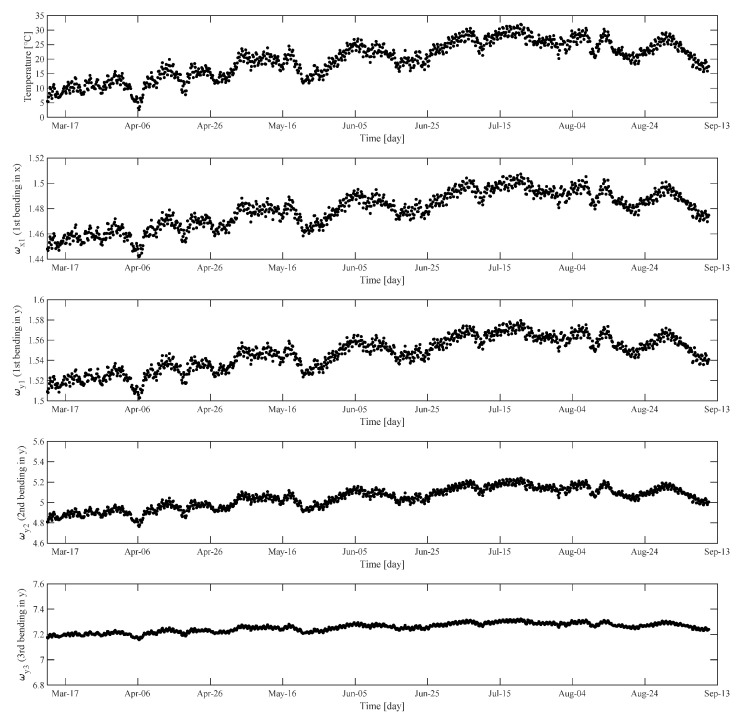
Measured data of temperature (T) and modal frequencies (${\bar{\omega }}_{x1}$, ${\bar{\omega }}_{y1}$, ${\bar{\omega }}_{y2}$, ${\bar{\omega }}_{y3}$ on different days.

Figure 6 shows measured data of temperature (T) and modal frequencies (${\bar{\omega }}_{x1}$, ${\bar{\omega }}_{y1}$, ${\bar{\omega }}_{y2}$, ${\bar{\omega }}_{y3}$) on different days.

The candidate model class of input-output relation ($S_{1}$) is identical to the model output $Q_{n}(b|\bar{S})$ of Equation (19). The candidate model classes of measurement error are introduced as follows: (1) $E_{spat,1}E_{temp,1}$ ($E_{spat,1}=E_{1}$ and $E_{temp,1}=E_{1}$); (2) $E_{spat,2}E_{temp,1}$ ($E_{spat,2}=E_{2}$ and $E_{temp,1}=E_{1}$); (3) $E_{spat,2}E_{temp,2}$ ($E_{spat,2}=E_{2}$ and $E_{temp,2}=E_{2}$). Finally, the universal set of all candidate model classes is $M=\{S_{1}E_{spat,1}E_{temp,1}$*,*$S_{1}E_{spat,2}E_{temp,1}$*,*$S_{1}E_{spat,2}E_{temp,2}\}$.

**Figure 7:**
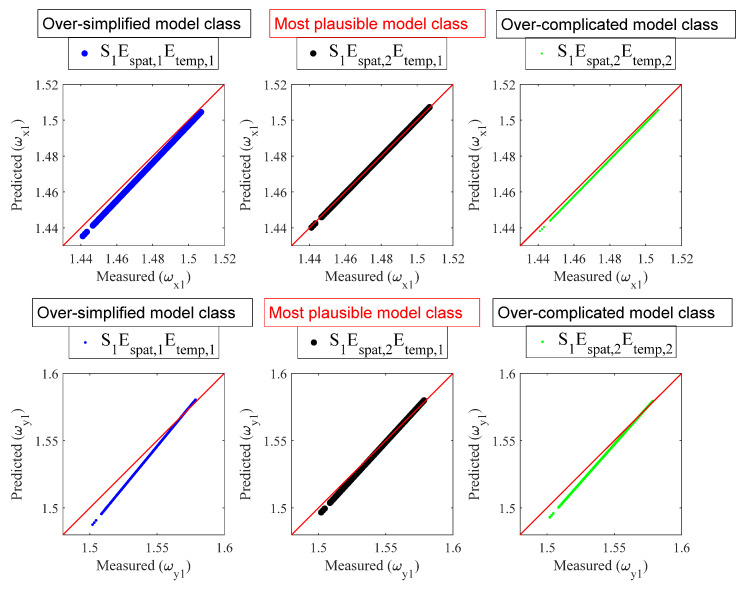
Measured and predicted values of $\omega_{x1}$ and $\omega_{y1}$ by different candidate model classes.

Table 6 shows model class selection results (modal frequency prediction). The most plausible model class is $E_{spat,2}E_{temp,1}$, indicating that the measurement errors are correlated in space but independent in time. The reason behind this result is explained as follows. On the one hand, for two modal frequencies at the same time step ($\omega_{x1,n}$ and $\omega_{y1,n}$), they are identified from the same set of vibration data, so it can be expected that they are statistically correlated, which is equivalent to $E_{spat,2}=E_{2}$. On the other hand, for the same modal frequency at different time steps ($\omega_{x1,n}$ and $\omega_{x1,n\prime }$ for n≠n′), the temporal distance between them is significantly large, so it can be expected that they are statistically independent, which is equivalent to $E_{temp,1}=E_{1}$. Table 7 shows parameter identification results (modal frequency prediction). Figure 7 shows the measured and predicted values of $\omega_{x1}$ and $\omega_{y1}$ by different candidate model classes. The upper and lower three subplots are for $\omega_{x1}$ and $\omega_{y1}$, respectively. The horizontal and vertical axes are for measured and predicted values, respectively. The 45-degree reference line represents that the predicted and measured values are identical. The performance of the most plausible model class ($S_{1}E_{spat,2}E_{temp,1}$ of two middle subplots) is superior to that of the oversimplified model class ($S_{1}E_{spat,1}E_{temp,1}$ of two left subplots), and is similar to that of the overcomplicated model class ($S_{1}E_{spat,2}E_{temp,2}$ of two right subplots). This reconfirms the result of Table 6. On the one hand, the likelihood value of $S_{1}E_{spat,2}E_{temp,1}$ is higher than that of $S_{1}E_{spat,1}E_{temp,1}$. On the other hand, the likelihood values of $S_{1}E_{spat,2}E_{temp,1}$ and $S_{1}E_{spat,2}E_{temp,2}$ are similar, so, owing to the principle of model parsimony, a simpler model is to be preferred over unnecessarily complicated ones.

## 4. Conclusions

Measurement error is non-negligible and crucial in SHM data analysis. In many applications of SHM, measurement errors are statistically correlated in space and/or in time for data from sensor networks. This paper generalizes the form of correlated measurement error from spatial correlation only or temporal correlation only to spatial-temporal correlation. A new form of spatial-temporal correlation and the corresponding likelihood function are proposed, and multiple candidate model classes for the measurement error are constructed, including no correlation, spatial correlation, temporal correlation, and the proposed spatial-temporal correlation. Bayesian system identification is conducted to achieve not only the posterior probability density function (PDF) for the model parameters but also the posterior probability of each candidate model class for selecting the most suitable/plausible model class for the measurement error. Examples are presented with applications for model updating and modal frequency prediction under varying environmental conditions. It turns out that: (1) to analyze SHM data with correlated measurement error, it is essential to select the most plausible model of measurement errors based on a prescribed set of candidate model classes characterizing spatially and temporally correlated measurement error; (2) over-simplified or over-complicated model class for the correlated measurement error degrades the identification result; (3) the proposed Bayesian system identification is capable of analyzing SHM data with correlated measurement error without knowing the true correlation model and the associated parameters.

## Figures and Tables

**Table 1:** Prior PDF of parameters.

	$b_{j}^{K}$	$b_{j}^{L}$	$\tau_{\gamma }^{E_{spat,2}}$	$\tau_{\gamma }^{E_{temp,2}}$	$\tau_{\gamma }^{E_{spat,3}}$	$\tau_{\gamma }^{E_{temp,3}}$
Distribution	Gamma	Gamma	Gamma	Gamma	Gamma	Gamma
*MAPr*	1	1	0.5	0.5	10	0.2
COV	0.5	0.5	0.5	0.5	0.5	0.5

**Table 2:** Model class selection results (no damage case).

	$S_{1}E_{spat,1}E_{temp,1}$ (Over-Simplified)	$S_{1}E_{spat,2}E_{temp,2}$ (Most Plausible)	$S_{1}E_{spat,3}E_{temp,3}$ (Over-Complicated)
Log-likelihood	942.91	1004.71	991.37
Log-Ockham factor	−37.12	−44.20	−52.45
Posterior model probability	1.72 × 10−24	1.00	4.20 × 10^−10^

**Table 3:** Parameter identification results (no damage case).

Parameters	True Values	$S_{1}E_{spat,1}E_{temp,1}$ (Over-Simplified)	$S_{1}E_{spat,2}E_{temp,2}$ (Most Plausible)	$S_{1}E_{spat,3}E_{temp,3}$ (Over-Complicated)
$b_{1}^{K}$	1	1.022	1.003	1.002
$\mathrm{std}\left(b_{1}^{K}\right)$	-	0.016	0.010	0.013
$b_{2}^{K}$	1	0.980	0.996	0.994
$\mathrm{std}\left(b_{2}^{K}\right)$	-	0.021	0.014	0.020
$b_{3}^{K}$	1	1.032	1.015	1.039
$\mathrm{std}\left(b_{3}^{K}\right)$	-	0.025	0.017	0.024
$b_{4}^{K}$	1	0.987	0.993	0.992
$\mathrm{std}\left(b_{4}^{K}\right)$	-	0.021	0.013	0.019
$b_{5}^{K}$	1	0.981	0.996	0.988
$\mathrm{std}\left(b_{5}^{K}\right)$	-	0.022	0.014	0.020
$\tau_{0}$	1.853 × 10^−4^	1.490 × 10^−4^	1.642 × 10^−4^	1.474 × 10^−4^
$\mathrm{std}\left(\tau_{0}\right)$	-	8.899 × 10^−6^	1.449 × 10^−4^	1.090 × 10^−5^
$\tau_{\gamma }^{E_{spat,2}}$	0.5	-	0.403	-
$\mathrm{std}\left(\tau_{\gamma }^{E_{spat,2}}\right)$	-	-	0.080	-
$\tau_{\gamma }^{E_{temp,2}}$	0.5	-	0.559	-
$\mathrm{std}\left(E_{temp,2}\right)$	-	-	0.044	-

**Table 4:** Model class selection results (damaged case).

	$S_{1}E_{spat,1}E_{temp,1}$ (Over-Simplified)	$S_{1}E_{spat,2}E_{temp,2}$ (Most Plausible)	$S_{1}E_{spat,3}E_{temp,3}$ (Over-Complicated)
Log-likelihood	906.61	1032.51	987.70
Log-Ockham factor	−31.33	−41.75	−46.31
Posterior model probability	7.04 × 10^−51^	1.00	3.62 × 10^−22^

**Table 5:** Parameter identification results (damaged case).

Parameters	True Values	$S_{1}E_{spat,1}E_{temp,1}$ (Over-Simplified)	$S_{1}E_{spat,2}E_{temp,2}$ (Most Plausible)	$S_{1}E_{spat,3}E_{temp,3}$ (Over-Complicated)
$b_{1}^{K}$	0.8	0.818	0.809	0.810
$\mathrm{std}\left(b_{1}^{K}\right)$	-	0.014	0.008	0.013
$b_{2}^{K}$	1	0.997	0.998	0.997
$\mathrm{std}\left(b_{2}^{K}\right)$	-	0.030	0.016	0.027
$b_{3}^{K}$	0.8	0.812	0.815	0.823
$\mathrm{std}\left(b_{3}^{K}\right)$	-	0.024	0.015	0.026
$b_{4}^{K}$	1	1.011	1.010	1.040
$\mathrm{std}\left(b_{4}^{K}\right)$	-	0.028	0.016	0.029
$b_{5}^{K}$	1	0.981	0.989	0.967
$\mathrm{std}\left(b_{5}^{K}\right)$	-	0.027	0.015	0.024
$\tau_{0}$	1.585 × 10^−4^	1.389 × 10^−4^	1.078 × 10^−4^	1.206 × 10^−4^
$\mathrm{std}\left(\tau_{0}\right)$	-	1.134 × 10^−5^	2.490 × 10^−5^	1.266 × 10^−5^
$\tau_{\gamma }^{E_{spat,2}}$	0.5	-	0.365	-
$\mathrm{std}\left(\tau_{\gamma }^{E_{spat,2}}\right)$	-	-	0.150	-
$\tau_{\gamma }^{E_{temp,2}}$	0.5	-	0.435	-
$\mathrm{std}\left(E_{temp,2}\right)$	-	-	0.061	-

**Table 6:** Model class selection results (modal frequency prediction).

	$S_{1}E_{spat,1}E_{temp,1}$ (Over-Simplified)	$S_{1}E_{spat,2}E_{temp,1}$ (Most Plausible)	$S_{1}E_{spat,2}E_{temp,2}$ (Over-Complicated)
Log-likelihood	863.82	890.82	894.91
Log-Ockham factor	−37.48	−38.81	−42.31
Posterior model probability	4.79 × 10^−11^	0.92	0.08

**Table 7:** Parameter identification results (modal frequency prediction).

Parameters	$S_{1}E_{spat,1}E_{temp,1}$ (Over-Simplified)	$S_{1}E_{spat,2}E_{temp,1}$ (Most Plausible)	$S_{1}E_{spat,2}E_{temp,2}$ (Over-Complicated)
$b_{x1,1}$	1.429	1.434	1.432
$\mathrm{std}\left(b_{x1,1}\right)$	0.0019	0.0017	0.0015
$b_{x1,2}$	2.36	2.30	2.29
$\mathrm{std}\left(b_{x1,2}\right)$	0.1636	0.1402	0.0999
$b_{y1,1}$	1.479	1.489	1.485
$\mathrm{std}\left(b_{y1,1}\right)$	0.0018	0.0017	0.0017
$b_{y2,2}$	3.17	2.84	2.95
$\mathrm{std}\left(b_{y2,2}\right)$	0.1633	0.1405	0.0999
$b_{y2,1}$	4.712	4.719	4.715
$\mathrm{std}\left(b_{y2,1}\right)$	0.0072	0.0055	0.0050
$b_{y2,2}$	16.06	16.10	16.08
$\mathrm{std}\left(b_{y2,2}\right)$	0.811	0.796	0.785
$b_{y3,1}$	7.125	7.139	7.132
$\mathrm{std}\left(b_{y3,1}\right)$	0.0096	0.0087	0.0079
$b_{y3,2}$	5.54	5.57	5.55
$\mathrm{std}\left(b_{y3,2}\right)$	0.352	0.338	0.0329
